# A culture model for the assessment of phenylalanine neurotoxicity in phenylketonuria

**DOI:** 10.1007/s44164-021-00007-4

**Published:** 2022-01-27

**Authors:** Julian Kylies, Bianka Brunne, Gabriele M. Rune

**Affiliations:** 1 Institute of Neuroanatomy, University Medical Center Hamburg-Eppendorf, Martinistr. 52, 20246 Hamburg, Germany; 2 Institute of Cell Biology and Neurobiology, Charité Anatomy, Charité Universitätsmedizin Berlin, Charitéplatz 1 (intern: Virchowweg 6 CCO), 10117 Berlin, Germany

**Keywords:** Organotypic slice cultures, Hippocampus, PKU-adopted culture medium, Microglia, Synaptic proteins

## Abstract

**Objective:**

Phenylketonuria (PKU) is caused by a specific mutation of the phenylalanine hydroxylase (PAH) gene. The deficiency of PAH results in high phenylalanine levels (Phe), low tyrosine levels (Tyr), and reduced catecholamine neurotransmitters. The majority of PKU patients, if untreated, develop severe mental retardation. The specific contribution of high Phe and low Tyr levels in mental retardation is largely unknown. In this study, we used organic hippocampal slice cultures in an optimized medium as an adequate culture model to decipher the precise role of high Phe and low Tyr levels on synaptic and glial integrity in PKU. The hippocampus is closely related to learning and memory and reduced catecholamine neurotransmitter levels can be neglected since these neurotransmitters do not derive from the hippocampus. Cultures exposed to physiological concentrations of Phe were compared with cultures exposed to doses of Phe/Tyr, as in the cerebral fluid of PKU patients.

**Methods:**

Using capillary western blot analysis and immunohistochemistry, followed by quantitative image analysis, we tested the expression of various pre- and postsynaptic proteins (PSD95, synaptopodin, SNAP25, synaptophysin), glial cell markers (GFAP, Iba1, P2Y12, CD68, C3b), and the morphology of glial cells.

**Results:**

We found a downregulation of the postsynaptic protein PSD95 and the presynaptic protein SNAP25 in the presence of high/low Phe/Tyr levels after 3 weeks, which, then however, recovered after 6 weeks in culture. Furthermore, no change in the expression pattern of glial proteins was observed.

**Conclusion:**

Our results show that high Phe levels/low Tyr levels alone are unlikely to substantially contribute to mental retardation in PKU. The direct neurotoxic potency of high Phe/low Tyr concentrations is almost negligible since the effects are transient. The transient character in the presence of unchanged levels of high Phe/low Tyr points to a role of reduced catecholamine derivate neurotransmitters, rather than of high Phe/low Tyr levels in PKU.

## Introduction

Phenylketonuria is an autosomal recessive inborn error of L-phenylalanine (Phe) metabolism (for review, see [1]). In phenylketonuria (PKU), mutations of the phenylalanine hydroxylase (PAH) gene decrease the ability of PAH to convert Phe to tyrosine (Tyr). This conversion takes place in the liver, the only organ with PAH expression. Consequently, PAH deficiency results in increased Phe levels and decreased Tyr levels in the blood and brain. The decrease in Tyr levels, in particular, is even worse in the brain, as Phe and Tyr use the same L-type amino acid transporter (LAT1), which has a high affinity for Phe [2]. As a consequence, low Tyr levels are followed by reduced neurotransmitter biosynthesis, such as serotonin and dopamine. Clinically, patients, if untreated, develop severe intellectual disability, epilepsy, and behavioral and psychiatric problems. A worldwide neonatal screening has been successfully established to diagnose and identify carriers of PKU. Treatment of PKU is centered on dietary modifications to include a specifically manufactured diet that is low in protein and Phe free (for review, see [3, 4]).

Despite extensive biochemical characterization of the PKU disease and positive correlation between hyperphenylalaninemia and altered neurological, behavioral, and cognitive deficits, the pathophysiology of disability remains poorly understood. It has been suggested that competitive transport inhibition of neutral amino acids by high Phe levels at the blood brain barrier could inhibit protein synthesis in the brain [5] [6]. In addition, Phe as an essential amino acid is crucial for the synthesis of Tyr and its catecholamine derivate neurotransmitter such as dopamine, norepinephrine and epinephrine, and serotonin. PKU patients show reduced levels of these neurotransmitters [7]. Aside from the gray matter effects, neuropathology characterized by brain white matter abnormalities is often observed in PKU patients. There is a positive correlation between the severity of white matter alteration and the degree and duration of hyperphenylalaninemia [8]. With respect to intellectual disabilities of PKU patients, many studies have demonstrated neurotoxic properties of high levels of Phe in neuronal cultures, such as impaired survival and neurite outgrowth of rat cortical neurons in response to elevated Phe levels [9]. Malformation of dendritic trees with reduced lengths of neurites [10] was described, together with reduced synaptic density in the neocortex after exposure to high levels of Phe [11, 12]. It was shown that cytoskeleton proteins in the cortex are affected by Phe [12, 13]. Direct effects of increased Phe concentrations were demonstrated on glutamatergic signaling, specifically competition of l-phenylalanine with l-glycine at the glycine binding site of NMDA receptors and with l-glutamate at the glutamate binding site of AMPA receptors. Glutamatergic neurotransmission plays a key role in synaptic function, and the changes in neurotransmission may at least, in part, explain the pathophysiology associated with late or insufficiently treated PKU patients [14–17]. In all of these studies, primary dissociated cultures of cortex and hippocampus were used and the doses of Phe did not mirror the situation in PKU.

In recent years, PKU mouse models were increasingly used to assess PKU-related neurological effects [16, 18–23] [24] and they confirm, at least in part, previous findings from in vitro experiments and experiments in mice after treatment with Phe, by showing reductions in the number of axons, dendrites, and synapses and for the first time an imbalance of excitation/inhibition [25]. But there are also inconsistencies: for example, in the Pah^enu2^ mouse, synaptic pruning is delayed shortly after birth, resulting in an increase in synaptic density up to 12 weeks of age [16], while Phe treatment of postnatal hippocampal slice cultures with Phe resulted in a dramatic decrease in synapse number [12, 26]. In addition, no effect of Phe was seen on microglia in vitro [12], while microglia were affected in the Pah^enu2^ mouse [16, 21].

Due to the complex neuropathology in PKU patients, it would be useful to know to which degree single alterations in the cascades of Phe metabolism—high Phe levels, low Tyr levels, or altered catecholamine levels — contribute to mental retardation in PKU. Thus, in order to assess the neurotoxic potency of specifically high Phe levels and low Tyr levels, we used neonatal hippocampal slice cultures and an optimized culture medium.

Firstly, the hippocampus is functionally closely related to learning and memory. The slice cultures of this part of the brain allow the study of identified neuronal types and specific synaptic connections in the hippocampus, as was described by Frotscher et al. for the first time. Later on, del Rio and Soriano established this culture model as a tool for pharmacological screening [27, 28]. In this 3D-culture model, synaptic connectivity and synaptic functionality are preserved for weeks. In general, cells cultured as 3D models show features that are closer to in vivo conditions, concerning cell morphology, gene and protein expression, and cytoarchitecture (for review, see [29]). Furthermore, the neurons are cultivated together with glia and thereby offer the opportunity to control the effects of high Phe/low Tyr levels on microglia, which were seen to be affected in the PAH^enu2^ mouse [16].

Secondly, we compared slice cultures which were kept in an optimized culture medium. Phe/Tyr concentrations were supplemented in close analogy to the doses of the amino acids in PKU [26]. In previous studies, as listed above, the concentration of Phe was regularly too high, due to the fact that Phe level in commercially available culture media was ignored. Of note, commercially available neuronal culture media already contain Phe and Tyr levels high above physiological levels. For example, Phe concentration is about 400 µM in commercially available Neurobasal Medium, which would rather reflect serum levels in the range from 240 to 600 µM (in comparison a healthy state range is from 35 to 76 µM) [30].

Thirdly, in many studies related to PKU and mental retardation, the accumulation of Phe in the brain was considered to account for PKU-induced cerebral dysfunction rather than decreased neurotransmitter synthesis and release [31], but as both aspects are linked to each other it is difficult to investigate their individual contribution to the overall PKU phenotype. As catecholamines (dopamine, serotonin) reach the hippocampus from other brain areas [32–34], effects due to reduced levels of catecholamines are neglectable when using organotypic slice cultures from the hippocampus. This situation provides the opportunity to investigate the effects of high Phe and low Tyr decoupled from differences in neurotransmitter levels.

In contrast to previous studies, we found, if any, only a very mild effect on synaptic protein expression after 3 weeks. Most importantly, this effect was restored in the presence of unchanged doses of Phe/Tyr after 6 weeks of treatment. This finding strongly argues against a relevant neurotoxic effect of high Phe/low Tyr in PKU, which is a life-long disease. Synapse loss and reduced length of dendrites and axons [1] in dissociated cultures very likely result from Phe doses even far above the already high Phe levels in PKU. Similarly, no effects were found on glial protein expression. Thus, the effects on glia cells, as shown in the PAH^enu2^ mouse, do not appear to result from high Phe/low Tyr concentrations. Overall, using a cell culture system which really reflects PKU levels of Phe and Tyr reveals rather low impact on neurons and glial cells as compared to previous studies. This underlines the importance of carefully adapting cell culture conditions and sheds new light on the stand-alone impact of high Phe/low Tyr levels to the overall neurological phenotype of PKU.

## Material and methods

### Animals

For the present study, postnatal (P4–5) male and female C57BLl6/J mice were bred in accordance with the animal care guidelines of the University of Hamburg. All animal procedures were carried out in accordance with the German law on the use of laboratory animals (German Animal Welfare Act; project number ORG 996 by the “Amt für Verbraucherschutz, Lebensmittelsicherheit und Veterinärwesen”) and were performed at the University Medical Center Hamburg Eppendorf, Dept. of Neuroanatomy.

### Organotypic hippocampal slice cultures

Organotypic hippocampal slice cultures were prepared as previously described [35, 36], with some modifications. Briefly, C57BL6/J male and female littermates (postnatal days 4–5) were decapitated and both hippocampi were dissected and sliced perpendicular to their longitudinal axis (400 μm) using the McIlwain Tissue Chopper Standard (H. Saur Laborbedarf, Germany). Adjacent slices (sister slices) (2–3 doubles per hippocampus) were separated and single slices of each double were then transferred to separate membrane inserts (Millicell CM, 0.4-μm culture plate inserts, 30-mm diameter; Merck Millipore) and subjected to different experimental conditions (i.e., one slice of each sister slice served as a control, whereas the corresponding slice was subjected to experimental treatment). Organotypic slices were cultivated in 6-well plates containing 1.2 ml culture medium. Slices were maintained at 37 °C in a humidified, CO_2_-enriched atmosphere for 21 days in vitro (21DIV), or 42 days in vitro (42DIV). The cell culture medium was changed every second day.

### Experimental design

Slices from hippocampi of one pup were equally distributed onto three inserts intended for the different experimental conditions (control group: physiological Phe/Tyr levels, PKU group: high Phe/lowTyr levels). To mimic a normal and high Phe microenvironment, different concentrations of Phe and Tyr were added to custom-made l-glutamine (Glut)-, l-phenylalanine (Phe)-, phenol red-, and l-tyrosine (Tyr)-free Neurobasal™ Medium supplemented with 2% B27, 2 mM GlutaMAX, and 1% PenStrep (ThermoFisher Scientific). Final concentrations for Phe and Tyr are given in Table 1.

**Table 1:** Final concentrations for Phe and Tyr

	L-Phenylalanine (mM)	L-Tyrosine (mM)
Control medium	0.1	0.08
PKU medium	1	0.04

### Capillary western blot analysis

Organotypic hippocampal slice cultures (4–5 slices per sample) were homogenized in 60 μl RIPA buffer consisting of 150 mM NaCl, 1% (v/v) Nonidet P-40 (Sigma, Igepal, CA), 0.1% SDS (sodium dodecyl sulphate), 0.5% sodium deoxycholate, 5 mM EDTA, 50 mM Tris, and pH 7.5, supplemented with a phosphatase and protease inhibitor cocktail (PhosSTOP and Complete EDTA-free Roche, Germany). Samples were homogenized using a tissue homogenizer (Precellys*® *24 homogenizer, Bertin Technologies) for 2 min at 5000 rpm. After incubation on ice for 30 min, the lysates were centrifuged 20 min at 13,000 rpm at 4 °C. Supernatants were stored at − 80 °C until further use.

Protein concentrations were determined using the Bio-Rad Protein Assay according to the manufacturer’s protocol. Capillary western blot analysis was performed using the Protein Simple Jess system (Protein Simple, San Jose, California, USA) and the 12–230 kDa Separation Module (ProteinSimple, SM-W001) according to the manufacturer’s instructions. Briefly, samples were diluted in 0.1 × sample buffer, combined with 5 × Fluorescent Master Mix, and denatured at 95 °C for 5 min. Finally, samples, blocking buffer, primary antibodies, HRP/NIR-conjugated secondary antibodies (Table 2), and chemiluminescent substrate were pipetted into the separation plate (pre-filled with Separation Matrix 2, Stacking Matrix 2, Split Running Buffer 2, and Matrix Removal Buffer). For detection, either the Anti-Rabbit (ProteinSimple, 042–206) or the Anti-Mouse Detection Module (ProteinSimple, 042–205), depending on the primary antibody, was used. Protein normalization was performed using the Protein Normalization Assay Module (Protein simple, AM-PN01). The resulting electropherograms were inspected to check whether automatic peak detection required any manual correction. Peak area calculations were performed by the Compass for Simple Western software (version 6.0) using the default Gaussian method.

**Table 2:** Primary antibodies used in the present study

Primary antibodies	Host	Supplier catalogue no	Dilution
PSD95	Rabbit	Genetex, GTX133091	1:20
Synaptopodin	Mouse	Acris, BM5086P	1:10
SNAP25	Rabbit	Abcam, ab4/455	1:90
Synaptophysin	Rabbit	Cell Signalling, D35E4	1:25
GFAP	Rabbit	Dako, Z0334	1:250
P2Y12	Rabbit	Invitrogen, 4H5L19	1:50
CD68	Mouse	Novus Biologicals, SPM130	1:50
C3b	Mouse	SantaCruz Biotechnology, A0820	1:50

### Immunofluorescence

Slices were fixed with 4% paraformaldehyde (PFA) (in phosphate-buffered saline (PBS)) for 1 h at room temperature (RT) and cryoprotected in 25% sucrose (in PBS) overnight at 4 °C. Slices were covered with embedding medium (Tissue-Tek® O.C.T.™ Compound; Sakura) and frozen on dry ice. Cryosections (14 µm) were mounted on glass slices, dried for 1 h at RT, and stored at − 20 °C until further use.

Tissue sections were thawed and air-dried for 1 h at RT. After washing with PBS containing 0.3% Triton (PBS-T), heat unmasking was performed using citrate buffer (pH 6). Unspecific binding sites were blocked with blocking buffer (2% normal horse serum, 1% BSA, 0.1 TritonX-100, and 0.05% Tween 20) for 1 h at RT. Incubation with the primary antibody was carried out in blocking buffer, overnight at 4 °C (Table 3). As secondary antibodies, Alexafluor 488 or 647 conjugated antibodies were used (1:500; Thermo Fisher Scientific, Germany), and applied for 2 h at RT. Cell nuclei were counterstained with DAPI (1:5000 Sigma-Aldrich). Sections were covered in fluorescence mounting medium (Dako, Germany).

**Table 3:** Primary antibody in blocking buffer for incubation

Primary antibodies	Host	Supplier catalogue no	Dilution	Secondary antibodies
PSD95	Rabbit	GeneTex, GTX133091	1:250	AlexaFluor 488 (1:500) Invitrogen A32731
Synaptophysin	Guinea pig	Synaptic Systems, 106,002	1:250	AlexaFluor 647 (1:500) Invitrogen A21450
IBA1	Guinea pig	Synaptic Systems, 234,004	1:300	AlexaFluor 488 (1:500) Invitrogen A11073
P2Y12R	Rabbit	ANASPEC, AS55043A	1:500	AlexaFluor 647 (1:500) Invitrogen A31573

### Image acquisition

Image acquisition of the sections was performed using the Axio Observer Z1 microscope, with ApoTome.2 module (Zeiss GmbH, Jena, Germany). Images were taken as Z-Stacks from the stratum radiatum of the CA1 and the stratum lucidum of the CA3 hippocampal region. Z-step was set to 0.2 µm for synapse analysis and 1 µm for microglia analysis.

For microglia analysis, a LCI Plan-Neofluar 25 × /0.8 Imm Korr DIC M27 objective was used with oil immersion; for synapse analysis a Plan-Apochromat 40 × /1.4 Oil DIC M27 objective was used.

### Microglia analysis— intensity, area-volume relationship, and density

To evaluate microglia activation, the staining intensity of Iba1 and P2Y12, as well as the area-volume relationship for every microglia, was measured in the stratum radiatum (CA1) using IMARIS software (Oxford Instruments, Ver. 9,7.2). Staining intensities of Iba1- and P2Y12- were measured as median intensities in every cell.

Area and volume reconstruction was carried out using both Iba1 and P2Y12 as reconstruction markers. The following parameters were used for Iba1 reconstruction: surface detail: 0.363 μm, absolute intensity: 469, exclusion of objects being less than 8327 voxels in size. The following parameters were used for P2Y12 reconstruction: surface detail: 0.363 μm, absolute intensity: 839, exclusion of objects being less than 7991 voxels in size. Only Iba1 and P2Y12 positive objects which contained a DAPI-stained nucleus were included in the data analysis, to exclude the possibility of measuring microglia fragments, rather than whole microglia cells. Microglia density analysis was carried out in the stratum radiatum (CA1) hippocampal region using FIJI software [37]. The region of interest was outlined in ImageJ using the polygon tool, and the area was measured. The paintbrush tool was used to mark each microglial cell to prevent double counting. Microglia were identified using the microglial cell markers Iba1 and P2Y12. Cells expressing both Iba1 and P2Y12 were labelled as double positive; cells expressing only Iba1 or P2Y12 were named Iba1 or P2Y12 single positive. Only microglia that had a DAPI-stained nucleus were used for analysis. Microglial density was calculated at the number of microglia/volume in μm^3^. Data were collected by a single, trained researcher blind to conditions.

### Synaptophysin, SNAP25, and PSD95 image analysis

An image field of a defined size (16 μm × 16 μm) was placed on the stratum radiatum of CA1 and the stratum lucidum of the CA3 region for synaptic protein analysis using the ROI (region of interest) tool, and staining intensities of synaptophysin, SNAP25, and PSD95 were measured using FIJI software (NCBI) [37].

### Statistical analysis

Parametric statistics were applied with simple western data (*N* = 10 animals for each time point) that met the Shapiro–Wilk criteria for normal distribution. Immunofluorescence data (*N* = 6 animals for each time-point) were analyzed by the non-parametric Mann–Whitney *U*-test. Statistical analysis was executed using IBM SPSS Statistics 22, and plots were prepared with GraphPad Prism 5.0 (GraphPad Software, San Diego, CA). Separated bar graphs showing mean with standard error (SEM) were used to graphically depict data. Differences were considered significant when *p* ≤ 0.05.

## Results and discussion

**Fig. 1:**
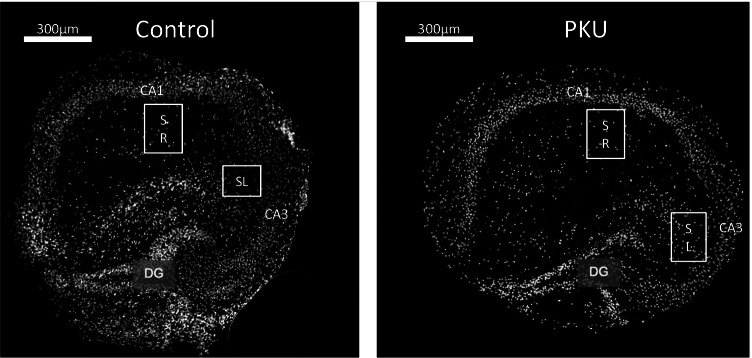
Example figures of hippocampal slice cultures showing typical layer arrangement in both control and PKU groups. No change in the structural integrity of the tissue was seen. For synapse analysis, pictures were taken from stratum radiatum (SR) CA1 and stratum lucidum (SL) CA3 hippocampal region. For microglia analysis, pictures were taken from the stratum radiatum (SR) CA1 (DG, dentate gyrus)

We had previously observed discrepant results regarding synaptic connectivity and glial protein expression in dissociated hippocampal neurons [12] in response to high levels of Phe and in the Pah^enu2^ mouse, the PKU mouse model [16], regarding synaptic connectivity and glial protein expression. In view of these discrepancies, we modified our experimental setup to mimic the PKU situation more closely. We used hippocampal slice cultures instead of dissociated neurons, which contain all types of glial cells in addition to all types of neurons, to maintain the in vivo structural organization. In addition, we used a custom-made Neurobasal Medium, delivered without Phe and Tyr, enabling us to adjust the level of these amino acids to meet the normal physiological brain concentration (0.08 mM Phe and 0.08 mM Tyr), or that of Pah^enu2^ mice (1 mM Phe and 0.04 mM Tyr). This regimen closely mimics, more closely than in our previous studies, the PKU situation. In both cases, control conditions with physiological concentrations of Phe and Tyr, as well as PKU conditions with elevated Phe and reduced Tyr levels, the morphology, and the typical hippocampal layer arrangement were well preserved in the slice cultures (Fig. 1).

In these hippocampal slice cultures, under the influence of defined doses of Phe/Tyr, we evaluated the expression of the postsynaptic density protein PSD95 [38] and synaptopodin [39–41]. PSD95 serves as a marker of postsynaptic spines of all types, and synaptopodin is a marker of selectively large, mature mushroom spines, the so-called memory spines. In addition, we studied synaptophysin and the synaptosomal-associated protein SNAP25 as presynaptic markers, which are involved in transmitter release. Synaptophysin is a constituent of the transmitter membrane [42] and SNAP25 is part of the presynaptic SNARE complex [43].

**Fig. 2:**
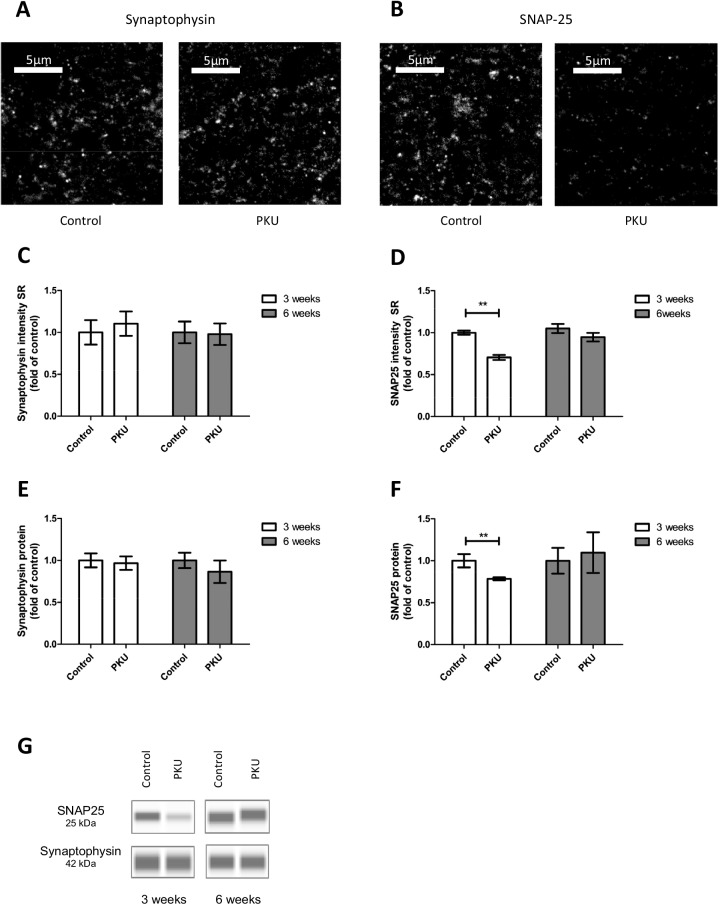
Immunoreactivity of presynaptic proteins in hippocampal slice cultures treated with PKU medium compared to control condition. Example figures of synaptophysin (**A**) and SNAP-25 (**B**) in the stratum radiatum (SR) CA1 hippocampal region of slices cultured for 3 weeks. **C** Quantitative evaluation of immunohistochemical staining of synaptophysin in stratum radiatum CA1 of slices cultured for 3 and 6 weeks. At both time points, there is no change in protein expression. **D** Quantitative evaluation of immunohistochemical staining of SNAP-25 in stratum radiatum CA1 of slices cultured for 3 and 6 weeks. In cultures exposed to PKU medium for 3 weeks, the protein is significantly downregulated. In slices cultured with PKU medium for 6 weeks, there is no change in protein expression. **E** and **F** Quantification of protein expression by capillary western blot analysis revealed no difference between synaptophysin in control cultures and cultures treated with PKU medium, but revealed a significant downregulation of SNAP-25 in cultures treated with PKU medium for 3 weeks. **G** Representative capillary western blots of synaptophysin and SNAP-25 (mean ± SEM, *N* = 10 animals, *P < 0.05, **P < 0.01, Mann–Whitney *U*-test or Student’s *t*-test)

**Fig. 3:**
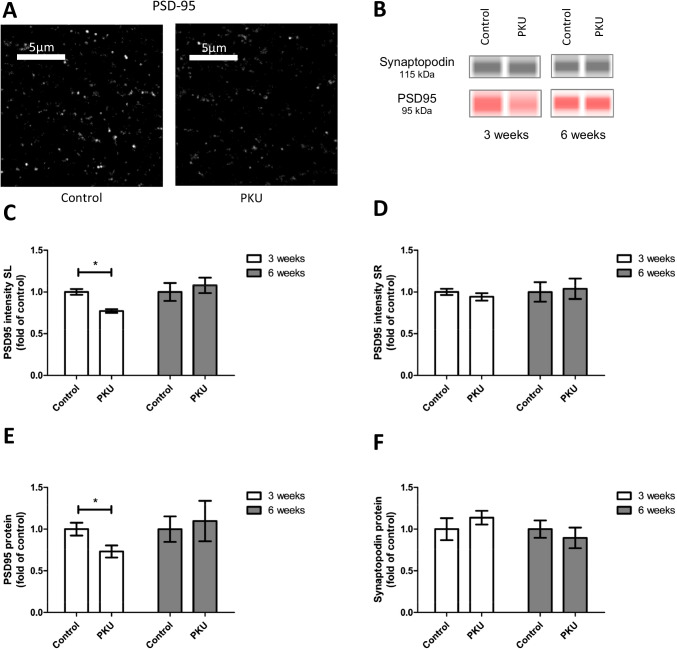
Immunoreactivity of postsynaptic proteins in hippocampal slice cultures treated with PKU medium compared to control condition. **A** Example figures of PSD-95 in the stratum lucidum (SL) CA3 hippocampal region of slices cultured for 3 weeks. **B** Representative capillary western blots of PSD-95 and synaptopodin proteins. **C** and **D** Quantitative evaluation of immunohistochemical staining of PSD-95 in stratum lucidum CA3 and stratum radiatum (SR) CA1 of slices cultured for 3 and 6 weeks. In slices cultured with PKU medium for 3 weeks, the protein is significantly downregulated in CA3, but not in CA1. In slices cultured with PKU medium for 6 weeks, there is no change in protein expression. **E** and **F** Quantification of protein expression by capillary western blot analysis revealed no difference between synaptopodin in control cultures and cultures treated with PKU medium, but revealed a significant downregulation of PSD-95 in cultures treated with PKU medium for 3 weeks (mean ± SEM, *N* = 10 animals, *P < 0.05, Mann–Whitney *U*-test or Student’s *t*-test)

SNAP 25 as a presynaptic marker was downregulated after 3 weeks, but this effect was restored after 6 weeks (Fig. 2). A similar time-dependent regulation was found with PSD95. The protein of the post synaptic membrane (Fig. 3) was reduced after 3 weeks, but restored after 6 weeks. In the case of SNAP25, this became evident using western blot analysis and also using image analysis of immunohistochemical staining. Downregulation of PSD95 was also seen in the western blots, and was confirmed by immunohistochemistry and subsequent image analysis, at least in the stratum lucidum of the CA3 hippocampal region (Fig. 3). Altogether, the findings confirm that, in fact, high Phe levels combined with low Tyr levels transiently and directly account for disturbing effects on synaptic connectivity and functionality. However, the effects were only seen in 3-week-old cultures, but not after 6 weeks of culture. This time dependency suggests a high vulnerability of the tissue within the first 3 weeks. It has been described that during this period of time the slices reorganize and synaptogenesis is completed, as in vivo [27, 28]. Once synaptogenesis has come to an end, synapses become resistant against high Phe/low Tyr levels. The recovery of the neurons after 6 weeks, however, strongly argues against primarily neurotoxic effects of high Phe/low Tyr, since PKU is a life-long disease. Above all, our findings contradict findings from earlier studies, including our own study, which showed severe neurotoxic effects in neuronal cultures, as listed in the “Introduction.” In our previous in vitro study on effects induced by high levels of Phe, we found that synapse density and dendritic length were dramatically reduced. Reduced cofilin expression and phosphorylation and reduced activation of the small GTPase Rac1 underlie these phenomena. In contrast, synapse integrity and functionality were only transiently disturbed in our modified experimental setup.

**Fig. 4:**
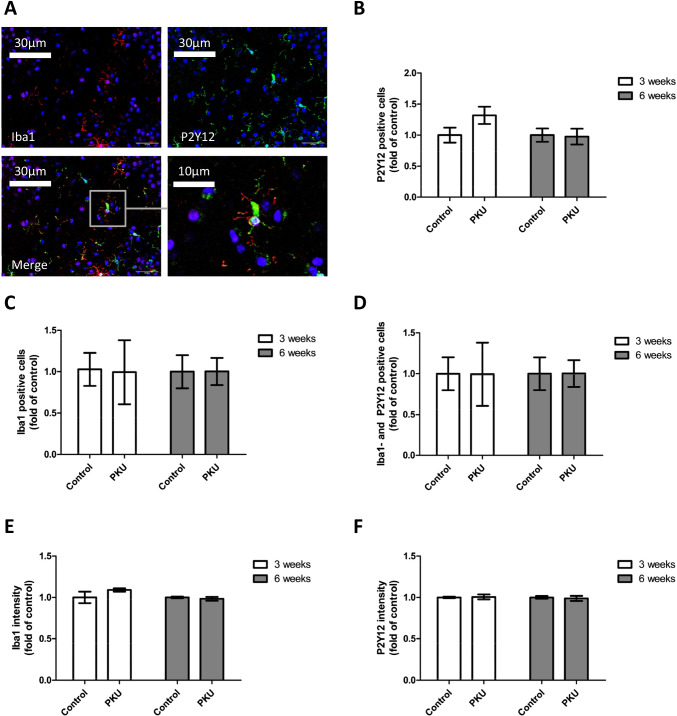
Immunoreactivity of Iba-1 and P2Y12 and microglia density in hippocampal slice cultures treated with PKU medium compared to control condition. **A** Example figures of Iba1 and P2Y12 immunoreactivity in the stratum radiatum (SR) CA1 region (PKU group), showing cell nuclei (DAPI, blue) and microglia (Iba1, red, P2Y12, green). **B**–**D** Quantitative evaluation of **B** P2Y12-positive, **C** Iba1-positive, and **D** P2Y12-Iba1-double positive cells in stratum radiatum CA1 hippocampal region revealed no difference in microglia numbers of slices cultured for 3 and 6 weeks. **E** and **F** Quantitative evaluation of Iba1 and P2Y12 immunoreactivity per cell by image analysis of immunohistochemical staining using IMARIS (mean ± SEM, *N* = 6 animals, *P < 0.05, Mann–Whitney *U*-test)

**Fig. 5:**
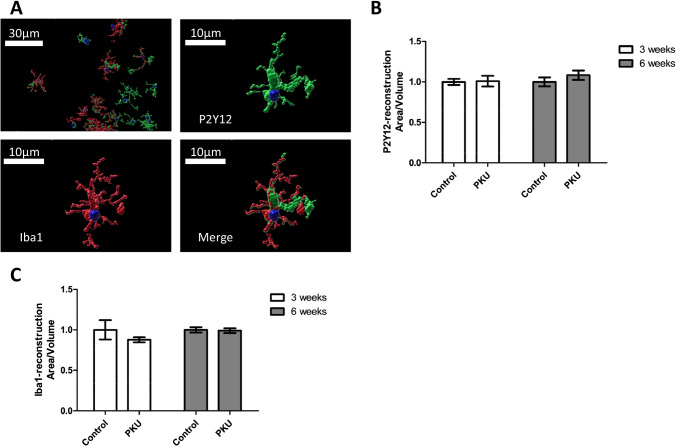
Microglia morphology analysis in hippocampal slice cultures treated with PKU medium compared to control condition. **A** Example figures of microglia 3D-reconstruction in stratum radiatum CA1 hippocampal region (PKU group) (DAPI, blue; Iba1, red; P2Y12, green). Quantitative evaluation of microglia area-volume relationship using **B** Iba1 and **C** P2Y12 staining as the reconstruction parameter revealed no difference between the control and PKU conditions, neither at 3 weeks nor at 6 weeks of culture (mean ± SEM, *N* = 6 animals, *P < 0.05, Mann–Whitney *U*-test)

**Fig. 6:**
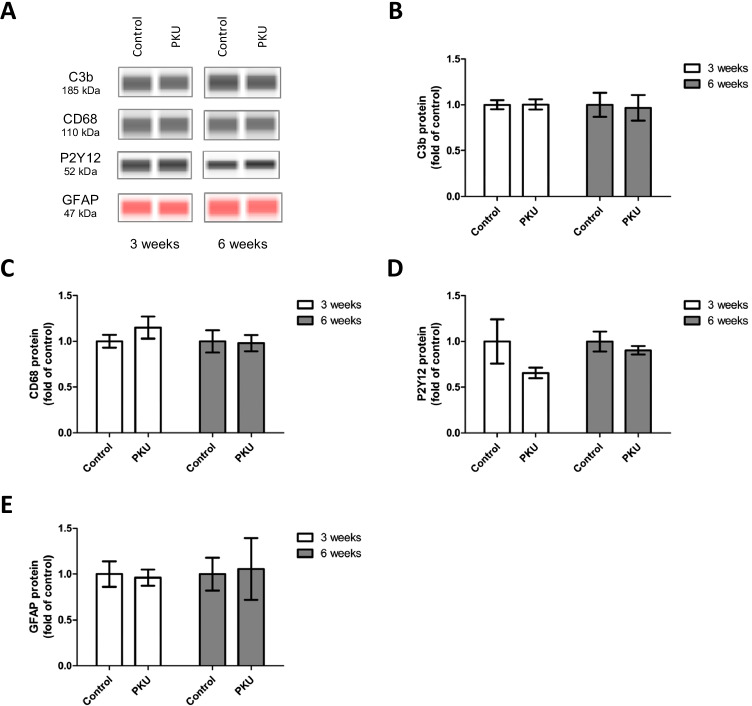
Glial cell markers in hippocampal slice cultures treated with PKU medium compared to control condition. **A** Representative capillary western blots of C3b, CD68, P2Y12, and GFAP. **B**–**E** Quantification of protein expression by capillary western blot analysis revealed no difference between the glial cell markers C3b, CD68, P2Y12, and GFAP in control cultures and cultures treated with PKU medium (mean ± SEM, *N* = 10 animals, *P < 0.05, Student’s *t*-test)

In order to assess toxic effects of high Phe/low Tyr levels on microglia, we studied the morphology of microglia present in the cultures. Activation of microglia goes along with changes in their morphology. In a quiescent state, they exhibit a ramified appearance and become circular as soon as they were activated. As commonly used markers, we used Iba1 and P2Y12 [44, 45]. No change was found in the morphology, neither in cell number nor in staining intensity in single cells (Figs. 4 and 5). In addition, we also studied complement C3b, which is expressed in neurons and participates in the recruitment of microglia, and CD68, a further microglia marker, and for comparative purposes P2Y12 by western blot analysis [46, 47]. No change in response to high Phe/low Tyr levels became evident (Fig. 6). Finally, we also tested GFAP, a protein specifically expressed in astrocytes, but the expression remained unchanged in our culture model (Fig. 6). Thus, the effects on glial cells that were seen in the PAH^enu2^ [16] mouse appear to result from other factors rather than from high Phe/low Tyr levels in PKU. In the Pah^enu2^ mouse, an established PKU mouse model, synaptic pruning after birth was delayed, resulting in increased synaptic density, despite the fact that synaptic transmission was heavily impaired on the pre- and postsynaptic sides [16]. In addition, we found decreased activity of microglia, as indicated by decreased Iba1 and C3 expression in the Pah^enu2^ mouse, which nicely fits in with the delay in synaptic pruning, since synaptic pruning is a function of microglia activity.

Amazingly, microglia were reported to be upregulated in 5-month-old Pah^enu2^ mice [21]. This finding was based on morphological criteria, since microglia retract their branches, and the size of the cell body increases as soon as they become activated. Accordingly, the ratio of cell body to cell size increases. De Groot and coworkers [21] concluded from their data that microglia are mildly upregulated in the Pah^enu2^ mouse. Unfortunately, they did not study the complement component C3, which is one of the factors that is required for microglia recruitment and activation [47–49]. We found mRNA downregulated in the 3-month-old Pah^enu2^ mice, which is consistent with reduced Iba1 immunoreactivity. Potentially, the discrepant findings in both studies [16, 21] result from the different ages of the animals under investigation. It could very well be that the postnatally delayed synaptic pruning, in combination with reduced microglia activity, ceases as the animals become older. Nevertheless, our new data reveal that increased Phe/lowTyr levels are unlikely to account for effects on microglia in the Pah^enu2^ mouse.

As a conclusion, for the first time, our data questions a sole role of high Phe/low Tyr levels in synaptic and glial integrity. Our in vitro model offers the opportunity to investigate the aspect of high Phe and low Tyr levels autonomously and to distinguish them from potential subsequent effects in the cascade to brain dysfunction. It appears that the processes which take place in the brain of PKU patients result from disturbances of Phe metabolism rather than from neurotoxic effects of high Phe/lowTyr levels themselves.

## Data Availability

Not applicable.
